# Magnolol Ameliorates Cisplatin-Induced Acute Kidney Injury with Activation of Nrf2-Associated Antioxidant Responses

**DOI:** 10.3390/cimb48010096

**Published:** 2026-01-17

**Authors:** Mi-Gyeong Gwon, Min Hui Park, Jaechan Leem

**Affiliations:** Department of Immunology, School of Medicine, Daegu Catholic University, Daegu 42472, Republic of Korea

**Keywords:** cisplatin, acute kidney injury, magnolol, oxidative stress, ferroptosis

## Abstract

Cisplatin (CDDP) is a cornerstone chemotherapeutic drug, yet its efficacy is frequently compromised by renal toxicity, primarily manifesting as acute kidney injury (AKI). Magnolol (MG) is a polyphenol from *Magnolia officinalis* and has been widely documented for its pronounced antioxidant and anti-inflammatory properties. This study evaluated the renoprotective effects of MG in a murine model of CDDP-induced AKI. Male C57BL/6 mice received MG (20 mg/kg) via daily intraperitoneal injection for four consecutive days, starting one day before a single CDDP injection. MG significantly reduced the serum concentrations of blood urea nitrogen and creatinine. Histopathological assessment revealed attenuated tubular damage and reduced expression of tubular injury markers. MG inhibited pro-inflammatory cytokines at both systemic and renal levels, alleviated endoplasmic reticulum stress, and suppressed activation of mitogen-activated protein kinase signaling pathways. Apoptotic damage was mitigated, as shown by the fewer TUNEL-positive cells and lowered expression of pro-apoptotic markers. In parallel, ferroptotic processes were alleviated through downregulation of pro-ferroptotic proteins and preservation of key antioxidant regulators. Importantly, MG restored nuclear factor erythroid 2-related factor 2 activity and upregulated downstream antioxidant effectors. These findings highlight the multi-targeted renoprotective actions of MG and support its possible utility as a therapeutic agent to prevent CDDP-induced renal injury.

## 1. Introduction

Cisplatin (CDDP) is a platinum-based chemotherapeutic agent widely used to treat various solid tumors, including ovarian, testicular, lung, and bladder cancers [1]. Despite its strong antitumor efficacy, the clinical application of CDDP is frequently limited by dose-dependent nephrotoxicity, with acute kidney injury (AKI) being one of its most serious adverse effects [2]. Therefore, elucidating the mechanisms underlying CDDP-induced AKI remains essential for identifying novel therapeutic avenues to preserve renal integrity.

CDDP-induced AKI is driven by a combination of interrelated pathological processes, including oxidative stress, inflammation, and renal tubular cell death [2]. CDDP preferentially accumulates in proximal tubular epithelial cells, where it promotes excessive generation of reactive oxygen species (ROS), leading to cellular damage and mitochondrial dysfunction [3]. This oxidative stress activates stress-responsive signaling pathways and triggers apoptotic cell death [4]. Concurrently, injured renal cells release damage-associated molecular patterns (DAMPs), which stimulate the production of pro-inflammatory cytokines such as tumor necrosis factor-α (TNF-α), interleukin-6 (IL-6), and interleukin-1β (IL-1β), thereby amplifying inflammatory injury within the kidney [5]. These processes act synergistically to exacerbate tubular damage and impair renal function.

Despite extensive research efforts, preventive strategies for CDDP-induced nephrotoxicity remain limited in clinical practice, with hydration being the primary supportive measure [6]. In this context, increasing attention has been directed toward naturally derived compounds with antioxidant and anti-inflammatory properties as potential renoprotective agents [7]. Magnolol (MG), a polyphenolic compound isolated from the bark of *Magnolia officinalis*, has been reported to exert diverse pharmacological effects, including robust antioxidant and anti-inflammatory activities [8,9]. Previous studies have shown that MG modulates key signaling pathways involved in oxidative stress and inflammatory responses, such as mitogen-activated protein kinase (MAPK) and nuclear factor erythroid 2-related factor 2 (Nrf2) signaling [10,11,12,13], suggesting its potential utility in renal protection.

Accordingly, the present study was designed to evaluate the renoprotective action of MG in a murine model of CDDP-induced AKI. We investigated whether MG administration could attenuate inflammatory responses, oxidative injury, and cell death within kidney tissue. Furthermore, given that CDDP-induced nephrotoxicity is accompanied by a robust systemic inflammatory response that further exacerbates renal injury, we assessed systemic biochemical and inflammatory markers to comprehensively evaluate the protective efficacy of MG. Clarifying the mechanisms underlying MG-mediated renoprotection may facilitate the formulation of improved therapeutic modalities for chemotherapy-associated AKI.

## 2. Materials and Methods

### 2.1. Animal Experiments

Male C57BL/6 mice at the age of seven weeks were supplied by HyoSung Science (Daegu, Republic of Korea). A one-week habituation period was provided to ensure that all animals stabilized under controlled conditions of 20–24 °C and 55% humidity. Lighting was automatically adjusted to a 12 h light and dark cycle. Once the habituation was complete, animals were assigned to four groups, each consisting of eight mice, using simple randomization based on a computer-generated random number sequence: (1) Control (no treatment); (2) MG; (3) CDDP; and (4) CDDP + MG (co-treatment). MG (20 mg/kg) was administered intraperitoneally once daily from Day 1 through Day 4. CDDP was delivered as a single intraperitoneal injection (20 mg/kg) on Day 2. MG and CDDP were acquired from Sigma-Aldrich (St. Louis, MO, USA; Cat# PHL89317 and P4394, respectively). In the CDDP + MG group, MG was administered 1 h prior to CDDP on Day 2 and then continued once daily through Day 4. The administration dose of MG in this study was selected in accordance with prior reports that established its protective capacity and lack of toxicity in C57BL/6 mice [14]. Mice were euthanized humanely 72 h after CDDP administration (on Day 5). Euthanasia was performed under deep anesthesia with Avertin (200 mg/kg; Sigma-Aldrich; Cat# T48402) administered via the intraperitoneal route. The anesthetic was freshly prepared as a 1.25% solution in 2-methyl-2-butanol. Following euthanasia, blood samples and kidney tissues were harvested for subsequent analyses. All animal procedures were approved by the Institutional Animal Care and Use Committee of Daegu Catholic University Medical Center (approval number: DCIAFCR-240102-34-Y).

### 2.2. Biochemical Analysis

Blood urea nitrogen (BUN) and serum creatinine levels were quantified using colorimetric detection kits (Invitrogen, Carlsbad, CA, USA; BUN: Cat# EIABUN; creatinine: Cat# EIASCR). In parallel, the concentrations of TNF-α, IL-6, and IL-1β in serum samples and kidney homogenates were quantified by enzyme-linked immunosorbent assay kits (R&D Systems, Minneapolis, MN, USA; Cat# MTA00B-1, M6000B-1, and MLB00C-1). To evaluate oxidative stress, lipid peroxidation was quantified by measuring malondialdehyde (MDA) levels (Sigma-Aldrich; Cat# MAK085). In addition, glutathione (GSH) levels were measured using reagents from Enzo Life Sciences (Farmingdale, NY, USA; Cat# ADI-900-160). All biochemical assays were conducted in strict accordance with the manufacturers’ instructions.

### 2.3. Histological Analysis and Immunohistochemistry (IHC)

Renal specimens were preserved in 10% neutral-buffered formalin and subsequently dehydrated through a stepwise ethanol series. After clearing with xylene, tissues were paraffin-infiltrated, sectioned, and mounted onto glass slides. The sections were stained with hematoxylin and eosin (H&E) as well as periodic acid-Schiff (PAS). The extent of tubular damage was analyzed in five random fields per section under 400× magnification. A standardized 5-point semi-quantitative grading system: 0 = no damage; 1 = <25%; 2 = 25–50%; 3 = 50–75%; and 4 = >75% of tubules showing injury [15]. For IHC staining, the slides underwent deparaffinization and rehydration followed by a formal antigen retrieval process. Following blocking, sections were exposed to rabbit polyclonal primary antibodies against either neutrophil gelatinase-associated lipocalin (NGAL; Invitrogen, Carlsbad, CA, USA; Cat# PA5-79590; 1:200) or 4-hydroxynonenal (4-HNE; Bioss, Woburn, MA, USA; Cat# BS-6313R; 1:200). Following several washes, a peroxidase-linked goat anti-rabbit IgG secondary antibody (Abcam, Cambridge, MA, USA; Cat# ab6721; 1:500) was applied. The extents of NGAL- and 4-HNE-positive staining were determined using the IMT i-Solution software (version 11.0, Coquitlam, BC, Canada). For this quantification, NGAL-positive areas were evaluated in five random fields at 400× magnification, whereas 4-HNE-positive signals were assessed at 200× magnification. All histopathological evaluations were performed by an experienced observer who was unaware of the experimental group allocation.

### 2.4. Terminal Deoxynucleotidyl Transferase dUTP Nick End Labeling (TUNEL) Staining

Apoptotic cell death was assessed using a TUNEL assay kit (Roche Diagnostics, Indianapolis, IN, USA; Cat# 11684795910). Paraffin-embedded kidney sections underwent deparaffinization and rehydration followed by a permeabilization step. The sections were then incubated with the TUNEL reaction solution in a humidified environment. Nuclear counterstaining was performed using 4′,6-diamidino-2-phenylindole (DAPI). Subsequently, fluorescent signals were imaged using a confocal laser-scanning microscope (Nikon, Tokyo, Japan). TUNEL-positive nuclei were quantified in five random fields per sample at 600× magnification. The quantification process was conducted by a blinded investigator.

### 2.5. Western Blotting

Total protein lysates were obtained from kidney tissue homogenates using a lysis reagent (Thermo Fisher Scientific, Waltham, MA, USA; Cat# 89901). To evaluate Nrf2 nuclear localization, nuclear-enriched protein fractions were prepared from kidney tissues using a nuclear isolation kit (Thermo Fisher Scientific; Cat# 78833), following the recommended protocol. Sample protein levels were determined by a bicinchoninic acid-based assay (Thermo Fisher Scientific; Cat# 23225). Equivalent quantities of protein were separated by gel electrophoresis and subsequently electro-transferred onto nitrocellulose membranes. To prevent non-specific binding, the membranes were blocked and subsequently probed with primary antibodies specifically targeting the proteins of interest under optimized conditions (Supplementary Table S1). Antibody specificity was verified by confirming the presence of a single distinct band at the expected molecular weight as indicated by the manufacturer and supported by prior validation in published literature. Following washing steps, the membranes were probed with peroxidase-linked goat anti-rabbit IgG (Cat# 7074; 1:2000) or horse anti-mouse IgG (Cat# 7076; 1:2000) secondary antibodies, both supplied by Cell Signaling Technology (Danvers, MA, USA). Signal detection was conducted using an enhanced chemiluminescence reagent (Thermo Fisher Scientific; Cat# 32106).

Protein band signals were measured by densitometric analysis using ImageJ software (version 1.53j, NIH, Bethesda, MD, USA), with the same analysis settings consistently applied to all samples. Although densitometric analysis was not performed in a blinded manner, quantification was conducted using objective, software-based methods to ensure analytical consistency and minimize investigator-dependent bias. For signaling analyses, phosphorylated proteins were detected first, after which membranes were stripped using a stripping buffer and reprobed for the corresponding total protein forms on the same membranes. The relative abundance of phosphorylated proteins was initially normalized to the expression levels of their corresponding total proteins, enabling direct assessment of phosphorylation status independent of changes in total protein expression. Subsequently, total protein levels for whole-cell lysates were standardized against glyceraldehyde-3-phosphate dehydrogenase (GAPDH) to ensure consistent protein loading, whereas lamin B1 served as the reference marker for the normalization of nuclear protein fractions.

### 2.6. Quantitative Polymerase Chain Reaction (qPCR)

Isolation of total RNA from kidney tissues was achieved using the TRIzol reagent (Sigma-Aldrich; Cat# T9424). Subsequently, reverse transcription was conducted to produce cDNA from the purified RNA templates. This process utilized the PrimeScript RT Reagent Kit (TaKaRa, Tokyo, Japan; Cat# RR037A) in strict accordance with the manufacturer-provided instructions. The reaction mixtures for PCR amplification were formulated to contain a cDNA input of 100 ng alongside 100 pM concentrations of both the forward and reverse primers. qPCR was conducted using a Thermal Cycler Dice Real Time System III (TaKaRa). The amplification protocol began with an initial heat activation step at 95 °C for 10 min, followed by 45 amplification cycles consisting of strand separation at 95 °C for 20 s, primer binding at 60 °C for 30 s, and DNA synthesis at 72 °C for 20 s. Fluorescence signals were monitored in real time using Power SYBR Green PCR Master Mix (Thermo Fisher Scientific; Cat# 4367660). Primer sequences are provided in Supplementary Table S2. To analyze the relative levels of gene expression, the 2^−ΔΔCT^ method was implemented. Data normalization was performed using GAPDH as the endogenous internal control to ensure analytical consistency across samples.

### 2.7. Statistical Analysis

Experimental results are presented as the mean ± standard error of the mean (SEM). All quantitative assessments were conducted using the GraphPad-Prism software (version 8.0, San Diego, CA, USA). To verify the distribution of the data, the Shapiro–Wilk test was initially utilized to evaluate normality. For data sets exhibiting a normal distribution, differences among multiple groups were evaluated using one-way analysis of variance (ANOVA), followed by Tukey’s test. When the data did not conform to a normal distribution, the Kruskal–Wallis test followed by Dunn’s test was implemented. A *p*-value of <0.05 was defined as the threshold for determining significant differences. Sample sizes varied depending on the experimental assay. In particular, Western blot analyses were performed using a subset of animals (*n* = 6) for which sufficient tissue quantity and protein quality were available, whereas other assays included all animals in each group (n = 8).

## 3. Results

### 3.1. MG Attenuates CDDP-Induced Renal Dysfunction and Histological Abnormalities

**Figure 1:**
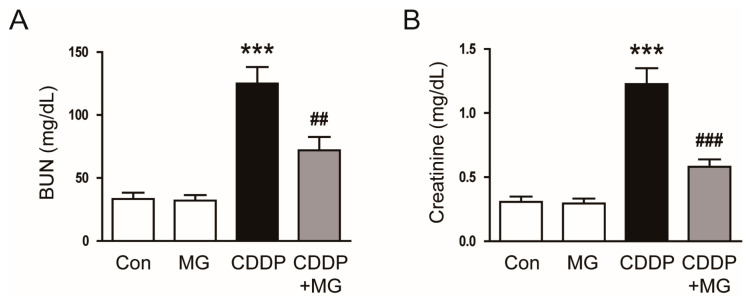
MG attenuates CDDP-induced renal dysfunction. (**A**) BUN and (**B**) serum creatinine levels in each group (*n* = 8). Data are expressed as mean ± SEM. *** *p* < 0.001 vs. Control; ^##^ *p* < 0.01, ^###^ *p* < 0.001 vs. CDDP.

To investigate how MG influences kidney function and tissue integrity under CDDP-induced nephrotoxic conditions, we first assessed key indicators of renal injury, including biochemical parameters and histological alterations. Administration of MG significantly attenuated CDDP-induced renal dysfunction, as demonstrated by pronounced declines in serum levels of BUN and creatinine concentrations (*p* = 0.008 for BUN; *p* < 0.001 for creatinine; Figure 1A,B).

**Figure 2:**
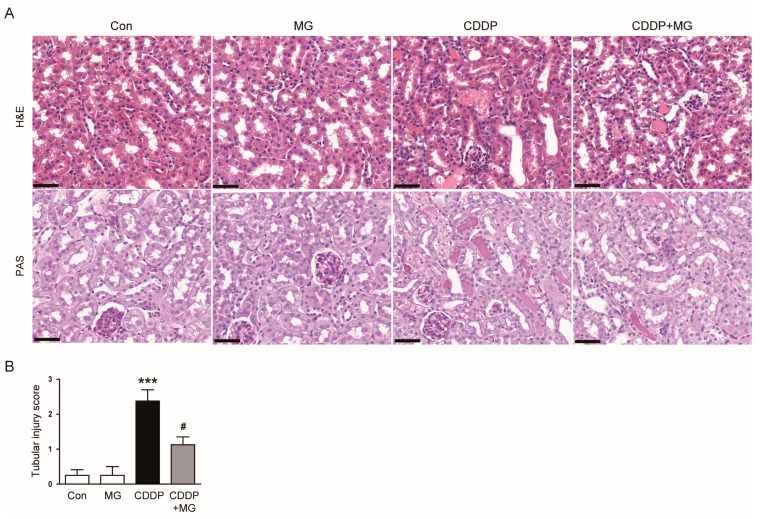
MG alleviates CDDP-induced histological kidney damage. (**A**) Representative histological images of kidney sections stained with H&E and PAS. Scale bars = 40 µm. (**B**) Tubular injury scores (*n* = 8). Data are expressed as mean ± SEM. *** *p* < 0.001 vs. Control; # *p* < 0.05 vs. CDDP.

Histological evaluation demonstrated that MG administration alleviated tubular injury, as indicated by a significantly reduced tubular injury score (*p* = 0.019; Figure 2A,B). Importantly, MG treatment alone did not affect renal function or histological morphology, indicating the absence of nephrotoxic effects (Figure 1A,B and Figure 2A,B).

**Figure 3:**
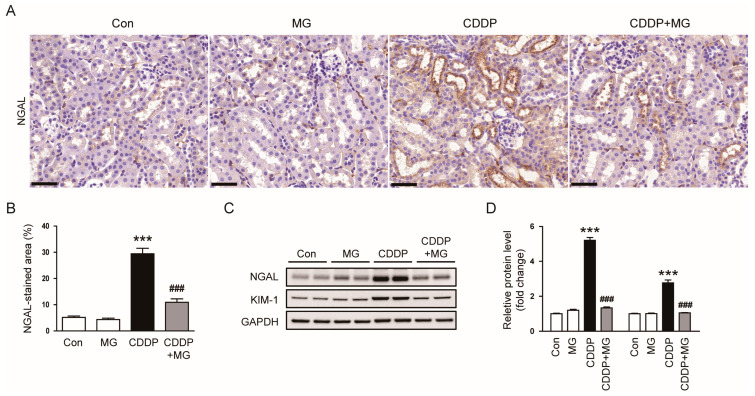
MG reduces CDDP-induced expression of tubular injury markers. (**A**) Representative IHC staining of NGAL. Scale bars = 40 µm. (**B**) Quantification of NGAL-positive areas (*n* = 8). (**C**,**D**) Western blotting and densitometric quantification of NGAL and KIM-1 in kidney tissues. (*n* = 6). Data are expressed as mean ± SEM. *** *p* < 0.001 vs. Control; ^###^ *p* < 0.001 vs. CDDP.

IHC staining showed a pronounced elevation in renal NGAL expression following CDDP exposure, which was substantially mitigated by MG administration (*p* < 0.001; Figure 3A,B). Consistently, MG markedly blunted the CDDP-induced upregulation of NGAL and kidney injury molecule-1 (KIM-1) protein levels (*p* < 0.001 for both; Figure 3C,D). Together, these data suggest that MG not only preserves renal function but also protects kidney structure from CDDP-induced damage.

### 3.2. MG Reduces CDDP-Induced Inflammatory Responses

**Figure 4:**
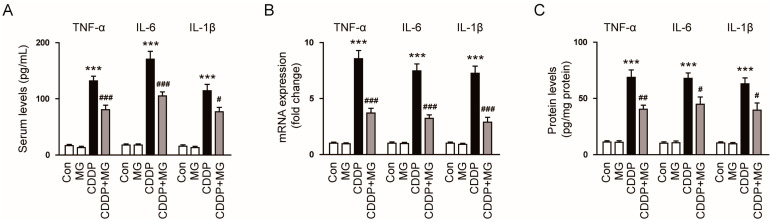
MG mitigates CDDP-induced inflammatory cytokine responses. (**A**) Serum concentrations of TNF-α, IL-6, and IL-1β (*n* = 8). (**B**) Relative transcript levels of these cytokines in kidney tissues (*n* = 8). (**C**) Corresponding protein expression levels in kidney tissues (*n* = 8). Data are expressed as mean ± SEM. *** *p* < 0.001 vs. Control; ^#^ *p* < 0.05, ^##^ *p* < 0.01, ^###^ *p* < 0.001 vs. CDDP.

Given the pivotal role of inflammation in CDDP-induced renal injury [2], we next examined whether MG modulates systemic and renal pro-inflammatory cytokine responses. Serum TNF-α, IL-6, and IL-1β abundances were substantially increased following CDDP treatment (Figure 4A). MG treatment significantly suppressed these elevated cytokine levels, indicating a potent anti-inflammatory effect (*p* < 0.001 for TNF-α and IL-6; *p* = 0.015 for IL-1β; Figure 4A). Consistently, the renal transcript levels of these cytokines were significantly upregulated following CDDP exposure and were effectively downregulated by MG administration (*p* < 0.001 for all; Figure 4B). A comparable trend was evident at the protein level, with cytokine levels increased following CDDP exposure and significantly reduced by MG treatment (*p* = 0.0025 for TNF-α; *p* = 0.03 for IL-6; *p* = 0.023 for IL-1β; Figure 4C). These results indicate that MG suppresses pro-inflammatory cytokines both systemically and locally in the kidney, reflecting its robust anti-inflammatory activity.

### 3.3. MG Suppresses CDDP-Induced Endoplasmic Reticulum (ER) Stress and MAPK Signaling Activation

**Figure 5:**
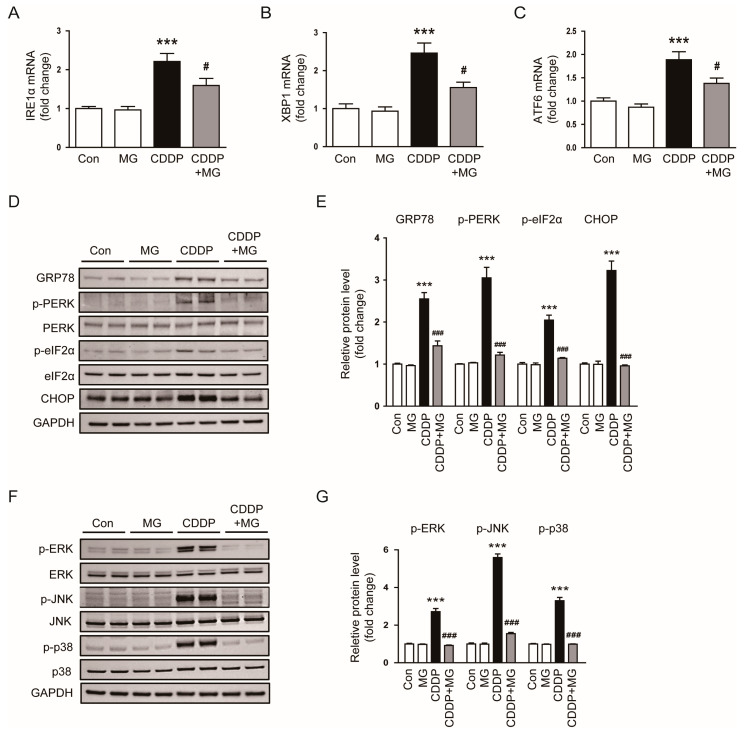
MG attenuates CDDP-induced ER stress and MAPK signaling activation. (**A**–**C**) Relative transcript levels of IRE1α, XBP1, and ATF6 in kidney tissues (*n* = 8). (**D**,**E**) Western blotting and densitometric quantification of GRP78, p-PERK, p-eIF2α, and CHOP in kidney tissues (*n* = 6). (**F**,**G**) Western blotting and densitometric quantification of p-ERK, p-JNK, and p-p38 (*n* = 6). The levels of phosphorylated proteins were first normalized to their respective total protein levels. Subsequently, total protein levels were normalized to GAPDH. Data are expressed as mean ± SEM. *** *p* < 0.001 vs. Control; ^#^ *p* < 0.05, ^###^ *p* < 0.001 vs. CDDP.

ER stress and MAPK signaling are crucial mediators of cellular stress and damage in response to toxic insults such as CDDP [16,17]. We investigated whether MG could modulate these pathways to attenuate cellular injury. To assess MG-mediated modulation of ER stress under CDDP treatment, transcripts of canonical ER stress markers were quantified by qPCR. Notably, transcripts of inositol-requiring enzyme 1α (IRE1α), X-box binding protein 1 (XBP1), and activating transcription factor 6 (ATF6) were robustly elevated following CDDP exposure, whereas MG treatment markedly downregulated their expression (*p* = 0.03 for IRE1α; *p* = 0.017 for XBP1; *p* = 0.028 for ATF6; Figure 5A–C). Consistently, CDDP markedly elevated the protein abundance of glucose-regulated protein 78 (GRP78), phosphorylated protein kinase R-like ER kinase (p-PERK), phosphorylated eukaryotic initiation factor 2α (p-eIF2α), and C/EBP homologous protein (CHOP) (Figure 5D,E). These changes were significantly attenuated by MG administration (*p* < 0.001 for all; Figure 5D,E). Furthermore, CDDP-triggered phosphorylation of extracellular signal-regulated kinase (ERK), c-Jun N-terminal kinase (JNK), and p38 was significantly suppressed following MG administration (*p* < 0.001 for all; Figure 5F,G). Collectively, these data indicate that MG mitigates CDDP-induced cellular stress responses by downregulating ER stress mediators and suppressing stress-activated MAPK signaling cascades.

### 3.4. MG Inhibits CDDP-Induced Apoptosis and Ferroptosis

**Figure 6:**
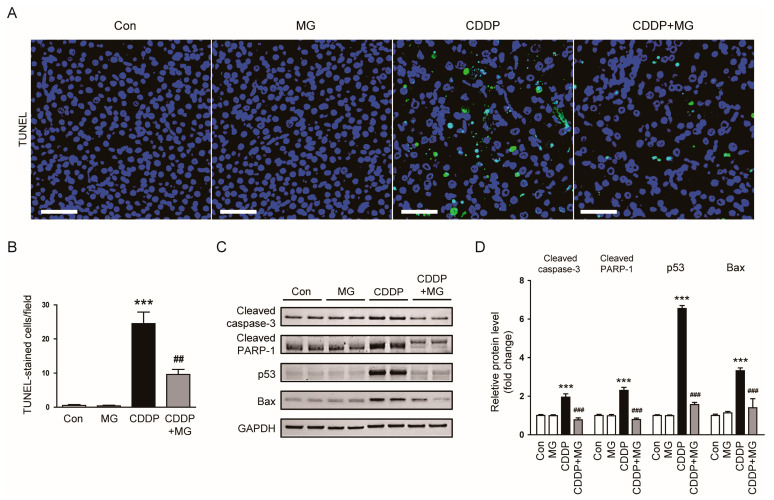
MG inhibits CDDP-induced apoptosis in kidney tissues. (**A**) Representative TUNEL-stained images. Scale bars = 50 µm. (**B**) TUNEL-positive cell counts per field (*n* = 8). (**C**,**D**) Western blotting and densitometric quantification of cleaved caspase-3, cleaved PARP-1, p53, and Bax (*n* = 6). Data are expressed as mean ± SEM. *** *p* < 0.001 vs. Control; ^##^ *p* < 0.01, ^###^ *p* < 0.001 vs. CDDP.

Apoptosis is a well-established form of programmed cell death that plays a central role in CDDP-induced nephrotoxicity [4]. To evaluate whether MG inhibits this pathway, we performed TUNEL staining. Extensive TUNEL-positive staining was observed in the kidneys of CDDP-exposed mice, whereas MG treatment effectively suppressed this increase (*p* = 0.002; Figure 6A,B). Consistently, CDDP elevated the expression of pro-apoptotic proteins, including cleaved caspase-3, cleaved poly (ADP-ribose) polymerase-1 (PARP-1), p53, and Bax (Figure 6C,D). These increases were markedly suppressed by MG administration (*p* < 0.001 for all; Figure 6C,D), supporting its anti-apoptotic effect.

**Figure 7:**
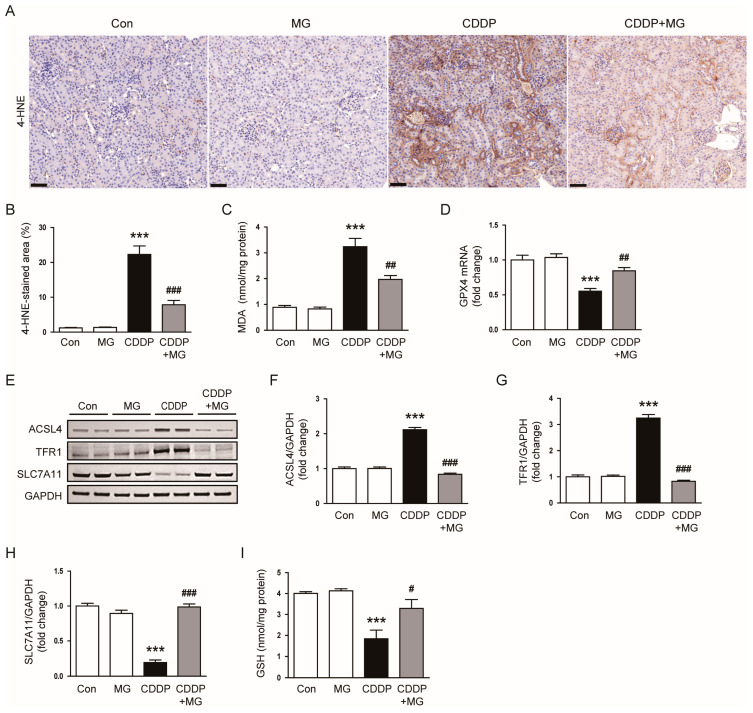
MG attenuates CDDP-induced oxidative stress and ferroptosis in kidney tissues. (**A**) Representative IHC staining of 4-HNE. Scale bars = 40 µm. (**B**) Quantification of 4-HNE-positive areas (*n* = 8). (**C**) Renal MDA levels (*n* = 8). (**D**) Relative transcript levels of GPX4 in kidney tissues (*n* = 8). (**E**–**H**) Western blotting and densitometric quantification of ACSL4, TFR1, and SLC7A11 (*n* = 6). (**I**) Renal GSH levels (*n* = 8). Data are expressed as mean ± SEM. *** *p* < 0.001 vs. Control; ^#^ *p* < 0.05, ^##^ *p* < 0.01, ^###^ *p* < 0.001 vs. CDDP.

In addition to apoptosis, ferroptosis is increasingly recognized as an important mechanism of CDDP-induced kidney injury, triggered by excessive lipid peroxidation and depletion of antioxidant defenses [18,19,20]. CDDP markedly increased oxidative stress in the kidney, as evidenced by enhanced 4-HNE staining and elevated MDA levels (Figure 7A–C). This oxidative burden was substantially reduced following MG treatment (*p* < 0.001 for 4-HNE; *p* = 0.003 for MDA; Figure 7A–C). Furthermore, CDDP downregulated the mRNA expression of glutathione peroxidase 4 (GPX4), a pivotal antioxidant enzyme involved in ferroptosis regulation, and MG significantly reversed this effect (*p* = 0.008; Figure 7D). At the protein level, CDDP upregulated the expression of acyl-CoA synthetase long-chain family member 4 (ACSL4) and transferrin receptor 1 (TFR1), both of which were significantly suppressed by MG (*p* < 0.001 for both; Figure 7E–G). In contrast, MG restored the CDDP-induced downregulation of solute carrier family 7 member 11 (SLC7A11), a cystine/glutamate antiporter critical for maintaining intracellular GSH levels (*p* < 0.001; Figure 7E,H). Additionally, MG replenished intracellular GSH levels, which were significantly depleted by CDDP exposure (*p* = 0.017; Figure 7I). Together, these data indicate that MG confers renoprotective effects by attenuating both apoptosis and ferroptosis, in parallel with mitigating oxidative stress and restoring redox homeostasis.

### 3.5. MG Restores CDDP-Suppressed Nrf2 Signaling Pathway in the Kidney

**Figure 8:**
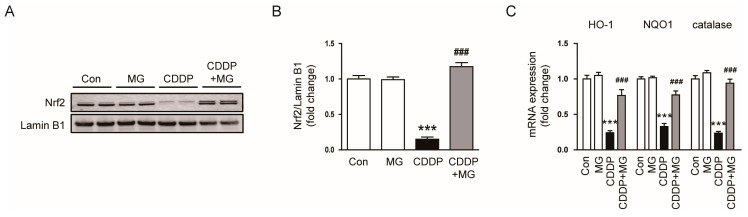
MG restores CDDP-suppressed Nrf2 signaling in the kidney. (**A**,**B**) Western blotting and densitometric quantification of nuclear Nrf2 (*n* = 6). (**C**) Relative transcript levels of HO-1, NQO1, and catalase (*n* = 8). Data are expressed as mean ± SEM. *** *p* < 0.001 vs. Control; ^###^ *p* < 0.001 vs. CDDP.

Given the role of the Nrf2 signaling pathway in maintaining redox homeostasis and combating oxidative injury [21,22], we evaluated whether MG could restore this pathway in cisplatin-challenged kidneys to enhance antioxidant defenses. CDDP exposure markedly diminished nuclear Nrf2 protein levels in the kidney (Figure 8A,B), indicating suppression of the Nrf2-mediated antioxidant defense pathway. This suppression was markedly reversed by MG treatment (*p* < 0.001; Figure 8A,B), suggesting that MG activates Nrf2 signaling under CDDP-induced oxidative stress. Furthermore, transcript levels of canonical Nrf2 targets, such as heme oxygenase-1 (HO-1), NAD(P)H quinone dehydrogenase 1 (NQO1), and catalase, were markedly decreased in the CDDP group but were effectively restored by MG treatment (*p* < 0.001 for all; Figure 8C). These data suggest that MG confers renal protection, at least in part, by restoring the Nrf2-driven antioxidant defense response suppressed by CDDP.

## 4. Discussion

Here, we demonstrated that MG effectively ameliorates CDDP-induced AKI by modulating multiple key pathogenic mechanisms. These protective effects were consistently observed at the histological, biochemical, and molecular levels. Notably, MG promoted the nuclear localization of Nrf2 along with upregulation of its downstream antioxidant targets. This restoration was accompanied by significant attenuation of CDDP-induced renal injury.

Oxidative stress is a primary pathogenic event in CDDP-induced AKI, occurring rapidly after drug accumulation in renal tubular epithelial cells [2,3]. Excessive ROS production, along with GSH depletion, causes oxidative damage to cellular macromolecules and disrupts redox homeostasis, ultimately triggering a cascade of events that results in cell death and inflammatory signaling [2,3]. CDDP administration markedly increased renal levels of lipid peroxidation byproducts and markedly decreased GSH levels, indicating a pronounced redox imbalance. MG treatment effectively reversed these changes, suggesting a potent antioxidant effect that stabilizes the oxidative environment in kidney tissue. Mechanistically, the antioxidant effects of MG are correlated with modulation of Nrf2-related regulatory processes. Nrf2 is a key transcription factor that maintains redox homeostasis by inducing the expression of antioxidant enzymes such as HO-1, NQO1, and catalase [21,22]. In our study, CDDP exposure suppressed nuclear accumulation of Nrf2 and downregulated its downstream targets, a pattern consistent with recent reports in CDDP-induced nephrotoxicity models [23,24,25,26]. Notably, MG administration restored nuclear Nrf2 protein levels and significantly upregulated HO-1, NQO1, and catalase mRNA expression, suggesting that MG reactivates this critical defense axis. This restoration of Nrf2 signaling may occur through direct ROS scavenging, thereby alleviating the inhibitory redox environment, or via activation of upstream kinases such as MAPK, which are known to enhance Nrf2 transcriptional activity [27]. These findings align with earlier studies in hepatic and neuroinflammatory models where MG enhanced Nrf2 activation and conferred protection against oxidative tissue injury [28,29,30]. The consistent activation of the Nrf2 pathway across organs highlights the systemic antioxidant capacity of MG and underscores its therapeutic potential in ROS-mediated pathologies. In the context of CDDP-induced nephrotoxicity, early modulation of redox imbalance is crucial, as ROS not only directly damages DNA, lipids, and proteins but also initiates downstream mechanisms including inflammation, ER stress, and various forms of cell death [3]. By targeting the oxidative insult at its source and reinstating the Nrf2-mediated antioxidant response, MG interrupts the initiating trigger of the pathogenic cascade. This upstream control may explain the broad downstream protective effects observed in this study, including reduced cytokine release, inhibition of cell death pathways, and preservation of renal function.

Inflammation is a central pathogenic factor in CDDP-induced nephrotoxicity, primarily triggered by oxidative stress and the release of DAMPs from injured renal tubular cells [2,5]. These signals promote immune cell activation and drive the release of pro-inflammatory mediators, further exacerbating renal injury. In this study, MG administration markedly reduced cytokine levels in serum and kidney tissues, indicating potent systemic and local anti-inflammatory effects. While the antioxidant properties of MG may account for part of this suppression, our findings suggest a broader regulatory role through its modulation of upstream signaling pathways. Notably, MG markedly inhibited CDDP-evoked ER stress, as reflected by reduced expression of canonical ER stress markers. Persistent ER stress is known to amplify inflammation by activating pro-inflammatory transcription factors and promoting the release of cytokines via IRE1α-mediated pathways [31,32]. By alleviating ER stress, MG likely prevents the initiation of this maladaptive inflammatory signaling cascade. Simultaneously, MG also suppressed the activation of key MAPKs, including ERK, JNK, and p38, which are central integrators of oxidative and inflammatory stimuli [33]. Thus, the inhibition of ER stress and MAPK signaling by MG does not merely alleviate cellular stress but also intersects crucial upstream nodes in the inflammatory network. This dual regulation is particularly relevant in the context of CDDP-induced nephrotoxicity, where inflammatory responses, oxidative imbalance, and renal cell death are tightly interconnected. By intervening at multiple levels of the pathophysiological cascade, including the suppression of ROS, alleviation of ER stress, and inhibition of stress-activated kinases, MG effectively interrupts the self-perpetuating cycle of inflammation and tissue injury in CDDP-induced nephrotoxicity.

Apoptosis and ferroptosis are both major contributors to tubular cell death in CDDP-induced nephrotoxicity, but they are governed by distinct signaling mechanisms [4]. Apoptosis is classically triggered by DNA damage, mitochondrial dysfunction, and ER stress, while ferroptosis arises from iron-mediated lipid peroxidation coupled with impaired antioxidant defenses. The observation that MG attenuates markers associated with both cell death pathways suggests that its renoprotective action is not limited to a single mechanism, but rather reflects a broad-spectrum cytoprotective effect. The anti-apoptotic effects of MG observed in this study are consistent with prior research demonstrating that MG attenuates apoptosis in a rodent model of renal ischemia–reperfusion injury [34]. In our study, MG markedly blunted the activation of caspase-3 and PARP-1 cleavage, further supporting its ability to interfere with the execution phase of apoptotic cell death. Additionally, by alleviating upstream stress signals such as ER stress and p38 MAPK activation, MG likely suppresses pro-apoptotic transcriptional programs initiated by CHOP and other stress-responsive mediators [35,36]. Ferroptosis has gained increasing attention as a form of regulated cell death that contributes to CDDP-induced renal injury, particularly through mechanisms involving GSH depletion and lipid peroxidation [18,19,20]. In our CDDP model, we observed biochemical and molecular hallmarks of ferroptosis that were substantially reversed by MG treatment. MG preserved the renal expression of GPX4 and SLC7A11, indicating that it helped maintain the antioxidant defenses essential for preventing lipid peroxidation. This anti-ferroptotic effect of MG is consistent with emerging findings from other biological contexts. Zhao et al. reported that MG upregulates Nrf2 and its downstream anti-ferroptotic genes in virally infected cells, thereby inhibiting ROS accumulation and ferroptotic damage [37]. Furthermore, the role of Nrf2 activation in conferring resistance to ferroptosis in CDDP-induced AKI is well established. Natural compounds such as melittin, amentoflavone, celastrol, and leonurine have been shown to activate Nrf2, enhance SLC7A11 and GPX4 expression, and reduce ferroptosis in CDDP-exposed kidneys [38,39,40,41]. Consistent with these findings, our data indicate that the ferroptosis-suppressing effects of MG may involve the Nrf2-SLC7A11/GPX4 axis.

Although the present findings are informative, there are inherent limitations to this study. First, all experiments were conducted exclusively in male mice, a design choice intended to minimize variability associated with the estrous cycle and female sex hormones, which are known to influence CDDP-induced renal injury [42]. Nevertheless, this design limits the broader applicability of the present findings, and additional investigations incorporating both sexes will be necessary to fully evaluate the renoprotective effects of MG. Another limitation of this study is the timing of MG administration. We adopted a preventive protocol in which MG was administered prior to CDDP injection to maximize the preconditioning of renal protective mechanisms. While this approach allowed us to confirm the renoprotective potential of MG, patients in clinical settings often receive protective agents after the initiation of chemotherapy. Therefore, further studies employing post-treatment therapeutic models are warranted to fully establish the clinical utility of MG in reversing established renal injury. Finally, while we observed changes in both MAPK signaling and Nrf2 pathways, the potential upstream regulation of Nrf2 by MAPK remains speculative. Since no direct causality experiments such as the use of specific inhibitors or gene silencing were performed, the exact relationship between these signaling nodes cannot be definitively established. Future studies utilizing loss-of-function approaches are required to confirm the regulatory mechanisms involved in MG-mediated Nrf2 activation.

## 5. Conclusions

The present findings demonstrate that MG provides pronounced renoprotective effects against CDDP-induced AKI through the modulation of multiple pathogenic mechanisms, including inflammatory signaling, oxidative damage, and regulated cell death pathways. These effects reflect MG’s multifaceted cytoprotective capacity, which is closely associated with enhanced Nrf2-driven antioxidant responses and reduced activation of stress-responsive signaling cascades. Collectively, these results position MG as a potential renoprotective agent in the setting of CDDP-based chemotherapy and provide a rationale for its continued evaluation beyond the current experimental framework.

## Data Availability

The original contributions presented in this study are included in the article/Supplementary Materials. Further inquiries can be directed to the corresponding author.

## Supplementary Materials

The supporting information can be downloaded at: https://www.mdpi.com/article/10.3390/cimb48010096/s1.

## References

[1] Ghosh S. (2019). Cisplatin: The first metal based anticancer drug. Bioorg. Chem..

[2] Tang C., Livingston M.J., Safirstein R., Dong Z. (2023). Cisplatin nephrotoxicity: New insights and therapeutic implications. Nat. Rev. Nephrol..

[3] McSweeney K.R., Gadanec L.K., Qaradakhi T., Ali B.A., Zulli A., Apostolopoulos V. (2021). Mechanisms of Cisplatin-Induced Acute Kidney Injury: Pathological Mechanisms, Pharmacological Interventions, and Genetic Mitigations. Cancers.

[4] Mapuskar K.A., Steinbach E.J., Zaher A., Riley D.P., Beardsley R.A., Keene J.L., Holmlund J.T., Anderson C.M., Zepeda-Orozco D., Buatti J.M. (2021). Mitochondrial Superoxide Dismutase in Cisplatin-Induced Kidney Injury. Antioxidants.

[5] Domingo I.K., Latif A., Bhavsar A.P. (2022). Pro-Inflammatory Signalling PRRopels Cisplatin-Induced Toxicity. Int. J. Mol. Sci..

[6] Volovat S., Apetrii M., Stefan A., Vlad C., Voroneanu L., Hogas M., Haisan A., Volovat C., Hogas S. (2023). Cisplatin and AKI: An ongoing battle with new perspectives—A narrative review. Int. Urol. Nephrol..

[7] Fang C.-Y., Lou D.-Y., Zhou L.-Q., Wang J.-C., Yang B., He Q.-J., Wang J.-J., Weng Q.-J. (2021). Natural products: Potential treatments for cisplatin-induced nephrotoxicity. Acta Pharmacol. Sin..

[8] Yuan Y., Zhou X., Wang Y., Wang Y., Teng X., Wang S. (2020). Cardiovascular Modulating Effects of Magnolol and Honokiol, Two Polyphenolic Compounds from Traditional Chinese Medicine-Magnolia Officinalis. Curr. Drug Targets.

[9] Lin Y., Li Y., Zeng Y., Tian B., Qu X., Yuan Q., Song Y. (2021). Pharmacology, Toxicity, Bioavailability, and Formulation of Magnolol: An Update. Front. Pharmacol..

[10] Chen H., Fu W., Chen H., You S., Liu X., Yang Y., Wei Y., Huang J., Rui W. (2019). Magnolol attenuates the inflammation and enhances phagocytosis through the activation of MAPK, NF-κB signal pathways in vitro and in vivo. Mol. Immunol..

[11] Chen J.H., Kuo H.C., Lee K.F., Tsai T.H. (2014). Magnolol protects neurons against ischemia injury via the downregulation of p38/MAPK, CHOP and nitrotyrosine. Toxicol. Appl. Pharmacol..

[12] Lu S.H., Hsu W.L., Chen T.H., Chou T.C. (2015). Activation of Nrf2/HO-1signaling pathway involves the anti-inflammatory activity of magnolol in *Porphyromonas gingivalis* lipopolysaccharide-stimulated mouse RAW 264.7 macrophages. Int. Immunopharmacol..

[13] Lu S.H., Chen T.H., Chou T.C. (2015). Magnolol Inhibits RANKL-induced osteoclast differentiation of raw 264.7 macrophages through heme oxygenase-1-dependent inhibition of NFATc1 expression. J. Nat. Prod..

[14] Liu Y., Lu M., Sun Q., Guo Z., Lin Y., Li S., Huang Y., Li Y., Fu Q. (2024). Magnolol attenuates macrophage pyroptosis triggered by *Streptococcus equi* subsp. *zooepidemicus*. Int. Immunopharmacol..

[15] Yu B., Jin L., Yao X., Zhang Y., Zhang G., Wang F., Su X., Fang Q., Xiao L., Yang Y. (2023). TRPM2 protects against cisplatin-induced acute kidney injury and mitochondrial dysfunction via modulating autophagy. Theranostics.

[16] Yan M., Shu S., Guo C., Tang C., Dong Z. (2018). Endoplasmic reticulum stress in ischemic and nephrotoxic acute kidney injury. Ann. Med..

[17] Kim E.K., Choi E.J. (2010). Pathological roles of MAPK signaling pathways in human diseases. Biochim. Biophys. Acta.

[18] Lai K., Chen Z., Lin S., Ye K., Yuan Y., Li G., Song Y., Ma H., Mak T.W., Xu Y. (2025). The IDH1-R132H mutation aggravates cisplatin-induced acute kidney injury by promoting ferroptosis through disrupting NDUFA1 and FSP1 interaction. Cell Death Differ..

[19] Li Y., Li K., Zhao W., Wang H., Xue X., Chen X., Li W., Xu P., Wang K., Liu P. (2023). VPA improves ferroptosis in tubular epithelial cells after cisplatin-induced acute kidney injury. Front. Pharmacol..

[20] Dong X.-Q., Chu L.-K., Cao X., Xiong Q.-W., Mao Y.-M., Chen C.-H., Bi Y.-L., Liu J., Yan X.-M. (2023). Glutathione metabolism rewiring protects renal tubule cells against cisplatin-induced apoptosis and ferroptosis. Redox Rep..

[21] Ma Q. (2013). Role of nrf2 in oxidative stress and toxicity. Annu. Rev. Pharmacol. Toxicol..

[22] Huang J., Zhao Y., Luo X., Luo Y., Ji J., Li J., Lai J., Liu Z., Chen Y., Lin Y. (2024). Dexmedetomidine inhibits ferroptosis and attenuates sepsis-induced acute kidney injury via activating the Nrf2/SLC7A11/FSP1/CoQ10 pathway. Redox Rep..

[23] Mapuskar K.A., Pulliam C.F., Zepeda-Orozco D., Griffin B.R., Furqan M., Spitz D.R., Allen B.G. (2023). Redox Regulation of Nrf2 in Cisplatin-Induced Kidney Injury. Antioxidants.

[24] Li H., Xu K., Mao W., Yu B., Liu Z., Huang F., Yang Z. (2025). Morroniside alleviates cisplatin-induced renal injury and gut dysbiosis via the gut–kidney axis and ferroptosis. Int. Immunopharmacol..

[25] Zhao L., Yue Z., Wang G., Qin J., Ma H., Tang D., Yin G. (2025). Smilax glabra roxb. alleviates cisplatin-induced acute kidney injury in mice by activating the Nrf2/HO-1 Signalling Pathway. Phytomedicine.

[26] Yalinbas-Kaya B., Tureyen A., Cesur S., Zemheri-Navruz F., Demirel H.H., Ince S. (2025). Iristectorin A Ameliorates Cisplatin-Induced Hepatorenal Injury in Mice Through Modulation of the Nrf2/HO-1 Signaling Pathway. J. Biochem. Mol. Toxicol..

[27] Bryan H.K., Olayanju A., Goldring C.E., Park B.K. (2013). The Nrf2 cell defence pathway: Keap1-dependent and -independent mechanisms of regulation. Biochem. Pharmacol..

[28] Liu X., Wang Y., Wu D., Li S., Wang C., Han Z., Wang J., Wang K., Yang Z., Wei Z. (2019). Magnolol Prevents Acute Alcoholic Liver Damage by Activating PI3K/Nrf2/PPARγ and Inhibiting NLRP3 Signaling Pathway. Front. Pharmacol..

[29] Kuo N.C., Huang S.Y., Yang C.Y., Shen H.H., Lee Y.M. (2020). Involvement of HO-1 and Autophagy in the Protective Effect of Magnolol in Hepatic Steatosis-Induced NLRP3 Inflammasome Activation In Vivo and In Vitro. Antioxidants.

[30] Tao W., Hu Y., Chen Z., Dai Y., Hu Y., Qi M. (2021). Magnolol attenuates depressive-like behaviors by polarizing microglia towards the M2 phenotype through the regulation of Nrf2/HO-1/NLRP3 signaling pathway. Phytomedicine.

[31] Garg A.D., Kaczmarek A., Krysko O., Vandenabeele P., Krysko D.V., Agostinis P. (2012). ER stress-induced inflammation: Does it aid or impede disease progression?. Trends Mol. Med..

[32] Kim S., Joe Y., Kim H.J., Kim Y.-S., Jeong S.O., Pae H.-O., Ryter S.W., Surh Y.-J., Chung H.T. (2015). Endoplasmic reticulum stress–induced IRE1α activation mediates cross-talk of GSK-3β and XBP-1 to regulate inflammatory cytokine production. J. Immunol..

[33] Kyriakis J.M., Avruch J. (2012). Mammalian MAPK signal transduction pathways activated by stress and inflammation: A 10-year update. Physiol. Rev..

[34] Tang C.-Y., Lai C.-C., Huang P.-H., Yang A.-H., Chiang S.-C., Huang P.C., Tseng K.-W., Huang C.-H. (2017). Magnolol Reduces Renal Ischemia and Reperfusion Injury via Inhibition of Apoptosis. Am. J. Chin. Med..

[35] Noh M.R., Kim J.I., Han S.J., Lee T.J., Park K.M. (2015). C/EBP homologous protein (CHOP) gene deficiency attenuates renal ischemia/reperfusion injury in mice. Biochim. Biophys. Acta.

[36] Francescato H.D., Costa R.S., da Silva C.G., Coimbra T.M. (2009). Treatment with a p38 MAPK inhibitor attenuates cisplatin nephrotoxicity starting after the beginning of renal damage. Life Sci..

[37] Zhao D., Guo X., Lin B., Huang R., Li H., Wang Q., Zeng Y., Shang Y., Wu Y. (2024). Magnolol against enterovirus 71 by targeting Nrf2-SLC7A11-GSH pathway. Biomed. Pharmacother..

[38] Zan H., Liu J., Yang M., Zhao H., Gao C., Dai Y., Wang Z., Liu H., Zhang Y. (2024). Melittin alleviates sepsis-induced acute kidney injury by promoting GPX4 expression to inhibit ferroptosis. Redox Rep..

[39] Zhang Y., Hu J., Zhang Y., Ci X. (2025). Amentoflavone protects against cisplatin-induced acute kidney injury by modulating Nrf2-mediated oxidative stress and ferroptosis and partially by activating Nrf2-dependent PANoptosis. Front. Pharmacol..

[40] Pan M., Wang Z., Wang Y., Jiang X., Fan Y., Gong F., Sun Y., Wang D. (2023). Celastrol alleviated acute kidney injury by inhibition of ferroptosis through Nrf2/GPX4 pathway. Biomed. Pharmacother..

[41] Hu J., Gu W., Ma N., Fan X., Ci X. (2022). Leonurine alleviates ferroptosis in cisplatin-induced acute kidney injury by activating the Nrf2 signalling pathway. Br. J. Pharmacol..

[42] Eshraghi-Jazi F., Nematbakhsh M. (2022). Sex Difference in Cisplatin-Induced Nephrotoxicity: Laboratory and Clinical Findings. J. Toxicol..

