# Supplementary material for: Magnolol Ameliorates Cisplatin-Induced Acute Kidney Injury with Activation of Nrf2-Associated Antioxidant Responses
Source: Curr Issues Mol Biol. 2026 Jan 17;48(1):96. doi: 10.3390/cimb48010096 (PMC12839844; doi:10.3390/cimb48010096)
Supplement: Supplementary file 1 [file cimb-48-00096-s001.zip › cimb-4049745-supplementary.pdf]

Magnolol Ameliorates Cisplatin-Induced Acute Kidney Injury with Activation of Nrf2-Associated Antioxidant Responses

Mi-Gyeong Gwon, Min Hui Park and Jaechan Leem

Supplementary Table S1. Primary antibodies for Western blot analysis

Protein	Supplier	Cat. No.	Species	Dilution
NGAL	Santa Cruz Biotechnology	sc-515876	Mouse	1:500
KIM-1	Abcam	ab47635	Rabbit	1:1000
GRP78	Cell Signaling Technology	3177	Rabbit	1:1000
p-PERK	Cell Signaling Technology	3179	Rabbit	1:1000
PERK	Santa Cruz Biotechnology	sc-377400	Mouse	1:500
p-eIF2α	Cell Signaling Technology	3597	Rabbit	1:1000
eIF2α	Santa Cruz Biotechnology	sc-133132	Mouse	1:500
CHOP	Invitrogen	MA1-250	Mouse	1:500
p-ERK	Cell Signaling Technology	9101	Rabbit	1:1000
ERK	Cell Signaling Technology	9102	Rabbit	1:1000
p-JNK	Cell Signaling Technology	9251	Rabbit	1:1000
JNK	Cell Signaling Technology	9252	Rabbit	1:1000
p-p38	Cell Signaling Technology	9211	Rabbit	1:1000
p38	Cell Signaling Technology	9212	Rabbit	1:1000
cleaved caspase-3	Cell Signaling Technology	9661	Rabbit	1:1000
cleaved PARP-1	Cell Signaling Technology	9541	Rabbit	1:1000
p53	Cell Signaling Technology	2524	Mouse	1:1000
Bax	Cell Signaling Technology	2772	Rabbit	1:1000
ACSL4	Santa Cruz Biotechnology	sc-365230	Mouse	1:500
TFR1	Abcam	ab269513	Mouse	1:1000
SLC7A11	Proteintech	32384-1-AP	Rabbit	1:500
Nrf2	Cell Signaling Technology	12721	Rabbit	1:1000
Lamin B1	Abcam	ab16048	Rabbit	1:1000
GAPDH	Cell Signaling Technology	5174	Rabbit	1:2000

Supplementary Table S2. Primers for qPCR

Gene	Primer sequence (5'→3')	Accession No.
TNF-α	F: ACTTCGGGGTGATCGGTCCCC<br>R: TGGTTTGCTACGACGTGGGCTAC	NM_013693
IL-6	F: TACCACTTCACAAGTCGGAGGC<br>R: CTGCAAGTGCATCATCGTTGTTC	NM_031168
IL-1β	F: CGCAGCAGCACATCAACAAGAGC<br>R: TGTCCTCATCCTGGAAGGTCCACG	NM_008361
IRE1α	F: GGTCCAATCGTACGGCAGTT<br>R: TCTCTCACAGAGCCACCTTTGTAG	NM_023913
XBP1	F: GAACACGCTTGGGAATGGACAC<br>R: AGAAAGGGAGGCTGGTAAGGAAG	NM_013842
ATF6	F: TGATGGCTGTCCAGTACACA<br>R: GCAGATGATCCCTTCGAAAT	NM_001081304
GPX4	F: CCTCTGCTGCAAGAGCCTCCC<br>R: CTTATCCAGGCAGACCATGTGC	NM_001037741
HO-1	F: AAGCCGAGAATGCTGAGTTCA<br>R: GCCGTGTAGATATGGTACAAGGA	NM_010442
NQO1	F: TGGAAGCTGCAGACCTGGTG<br>R: CCCTTGTCATACATGGTGGCATAC	NM_008706
Catalase	F: CACTGACGAGATGGCACACTTTG<br>R: TGGAGAACCGAACGGCAATAGG	NM_009804
GAPDH	F: TGGAAAGCTGTGGCGTGAT<br>R: TGCTTCACCACCTTCTTGAT	NM_001289726
